# Supplementary material for: Antiviral Activities of Ethyl Pheophorbides a and b Isolated from Aster pseudoglehnii against Influenza Viruses
Source: Molecules. 2022 Dec 21;28(1):41. doi: 10.3390/molecules28010041 (PMC9822050; doi:10.3390/molecules28010041)
Supplement: Supplementary file 1 [file molecules-28-00041-s001.zip › molecules-2095742-supplementary.pdf]

Supplementary metarials

Antiviral Activities of Ethyl Pheophorbides a and b Isolated from *Aster pseudoglehnii* against Influenza Viruses

Contents

Figure S1. HR-MS spectrum of compound **1**
Figure S2. ^{1}H-NMR (500 MHz, $CDCl_3$) spectrum of compound **1**
Figure S3. ^{13}C-NMR (125 MHz, $CDCl_3$) spectrum of compound **1**
Figure S4. LC-ESI-MS spectrum of compound **2**
Figure S5. ^{1}H-NMR (500 MHz, $CDCl_3$) spectrum of compound **2**
Figure S6. ^{13}C-NMR (500 MHz, $CDCl_3$) spectrum of compound **2**
Figure S7. Cytotoxicity of ethyl pheophorbides a and b against MDCK cells

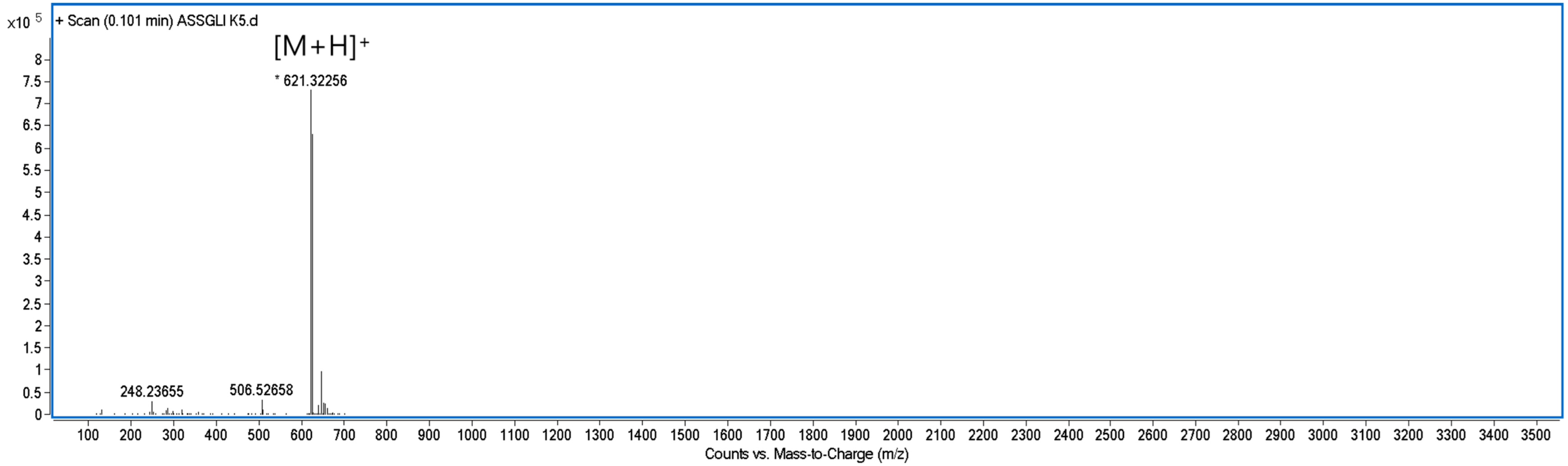

Figure S1. HR-MS spectrum of compound **1**

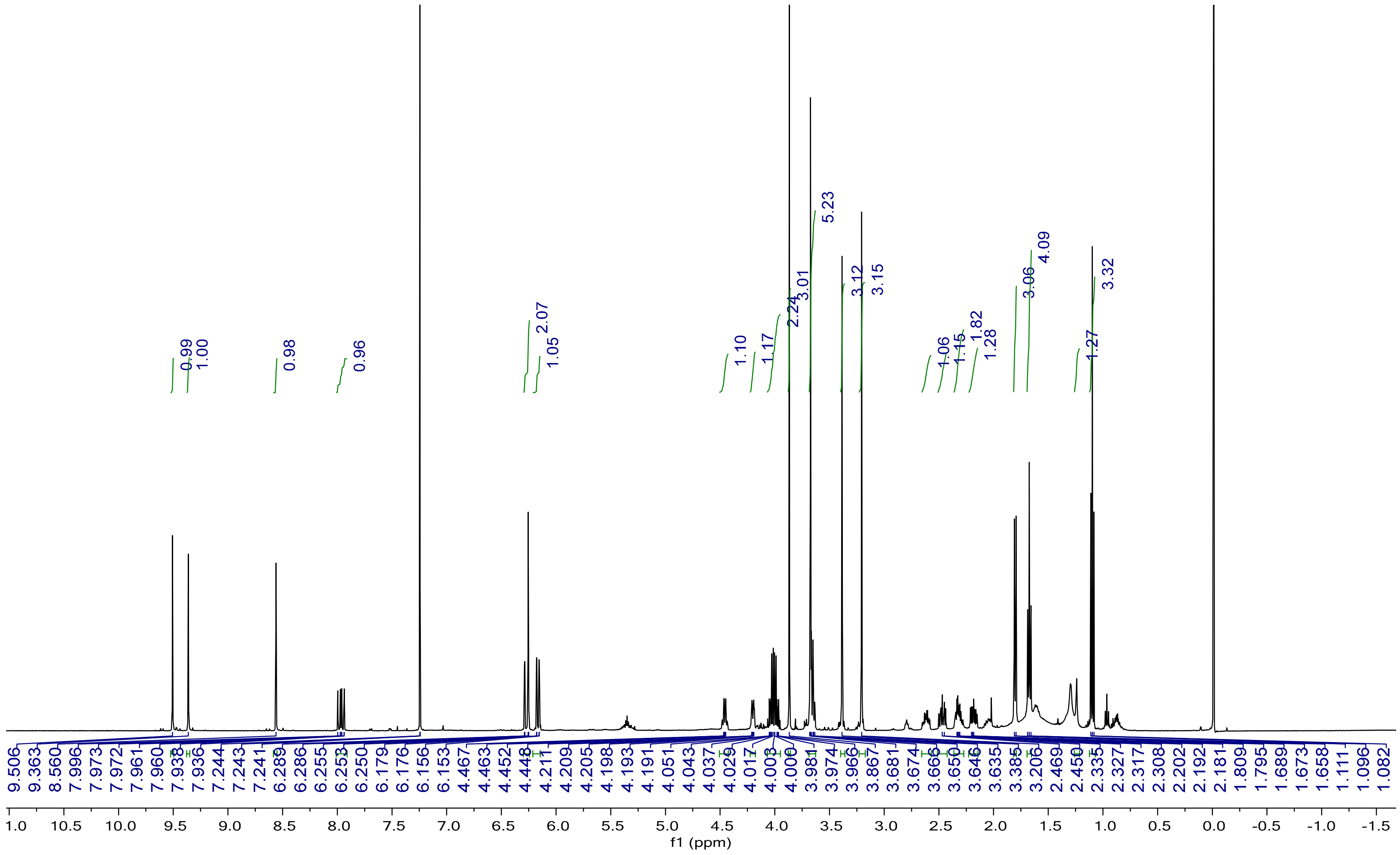

Figure S2. ^{1}H-NMR (500 MHz, $CDCl_3$) spectrum of compound **1**

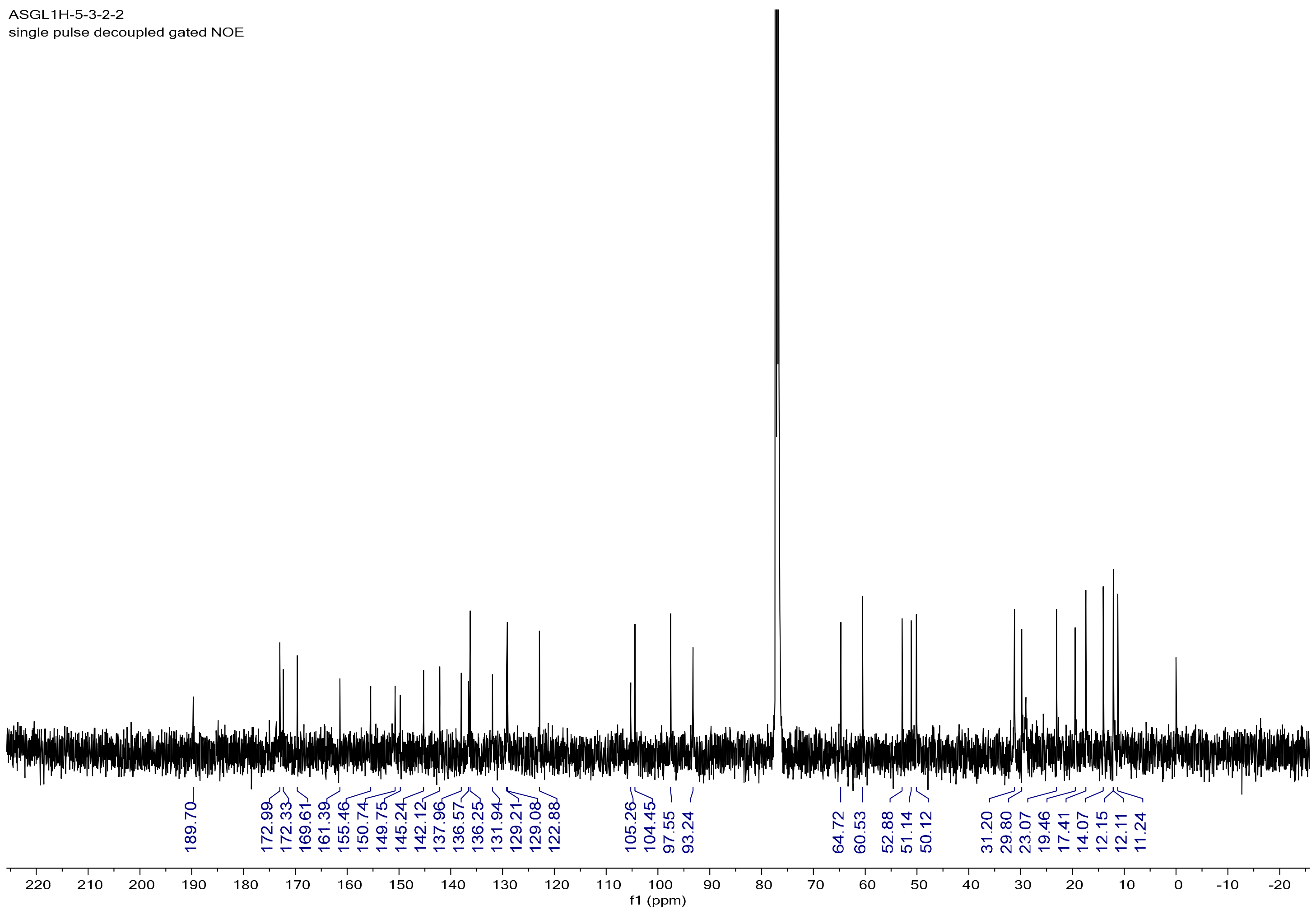

Figure S3. ^{13}C-NMR (125 MHz, $CDCl_3$) spectrum of compound **1**

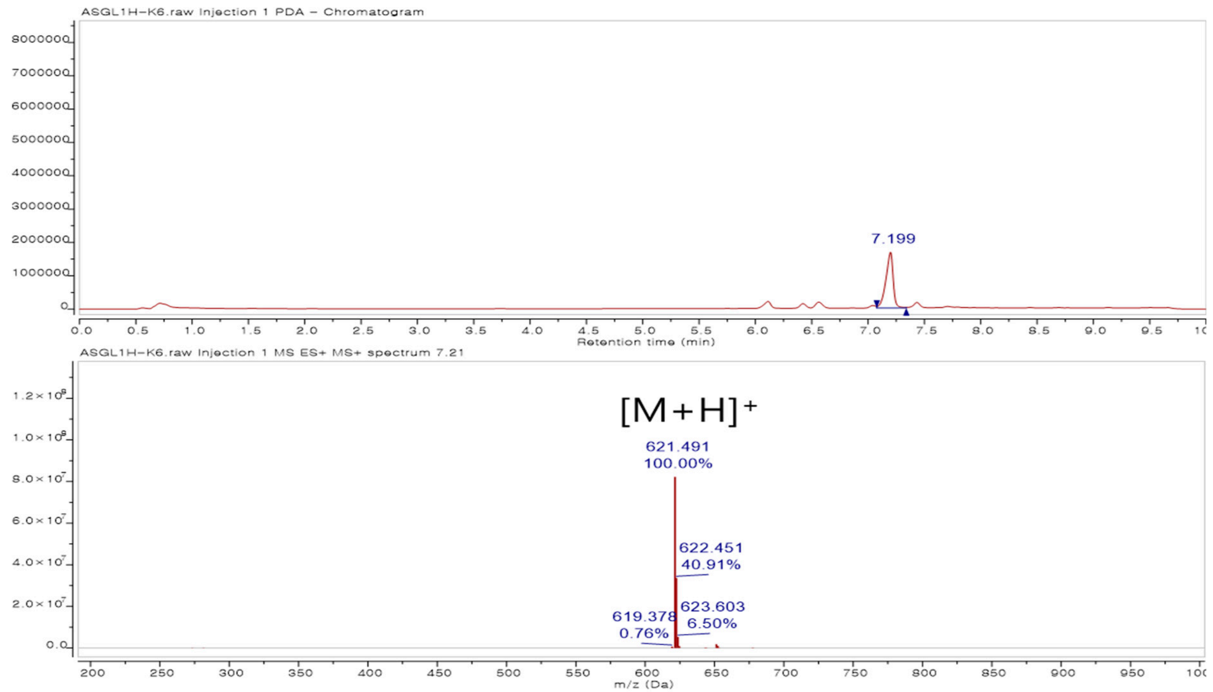

Figure S4. LC-ESI-MS spectrum of compound **2**

LC-ESI-MS was performed using the Waters ACQUITY UPLC system and Waters Micromass Quattro micro API (Waters, Milford, Massachusetts, USA) with an ACQUITY UPLC BEH C18 column (2.1× 50 mm i.d. 1.7 μm, Waters, Milford, Massachusetts, USA). The mobile phase consisted of water containing 0.1 % formic acid (solvent A) and acetonitrile containing 0.1 % formic acid (solvent B) with gradient elution at a flow rate of 0.2ml/min as follows: 75 % B (0-0.5 min); 75% to 90 % B (0.5-9.5); 75% B (9.5-10 min). The column was equilibrated under initial conditions for 1 min before the next injection. The injection volume was 3 μl and column temperature was 25°C.

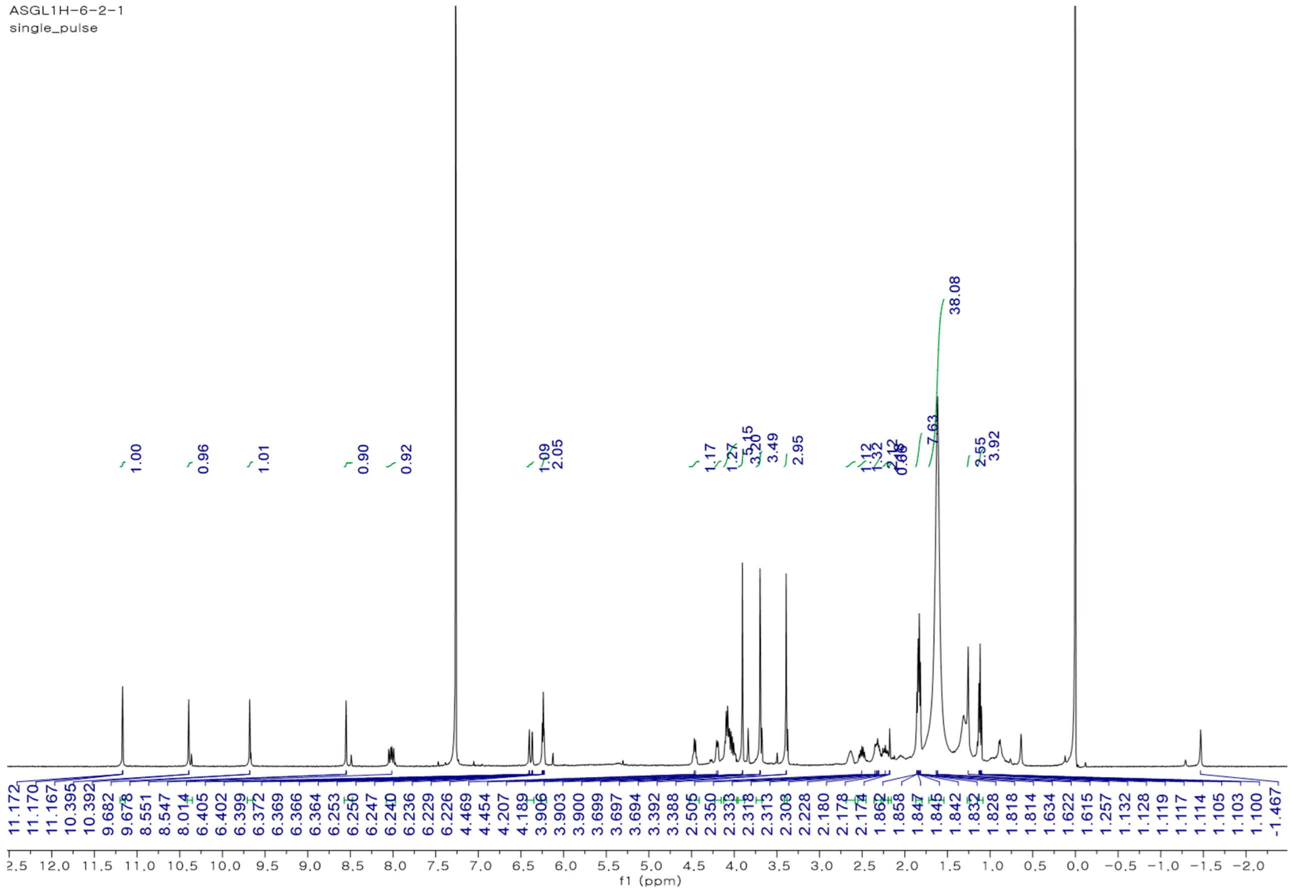

Figure S5. ¹H-NMR (500 MHz, $CDCl_3$) spectrum of compound **2**

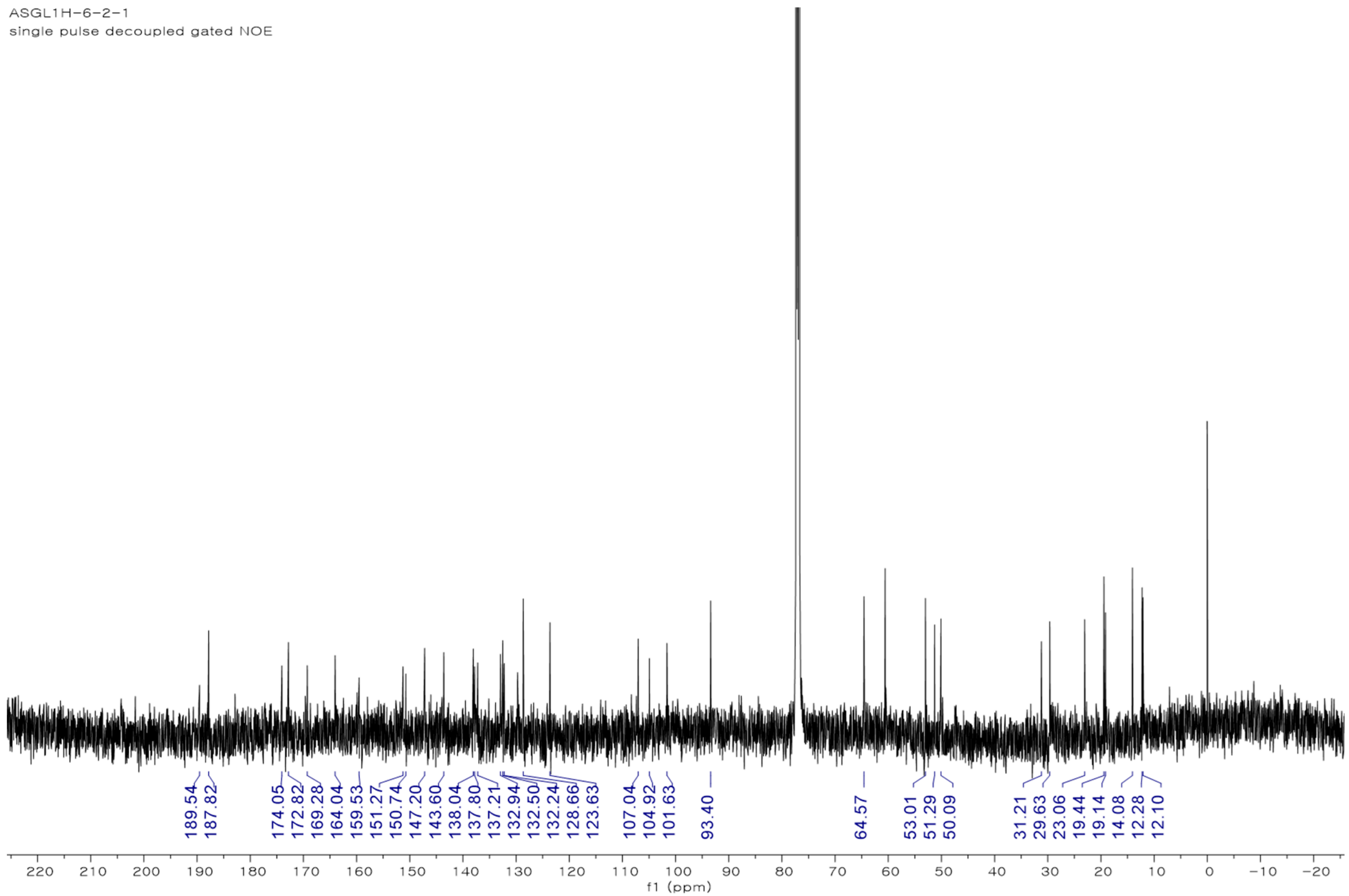

Figure S6. ^{13}C-NMR (500 MHz, $CDCl_3$) spectrum of compound **2**

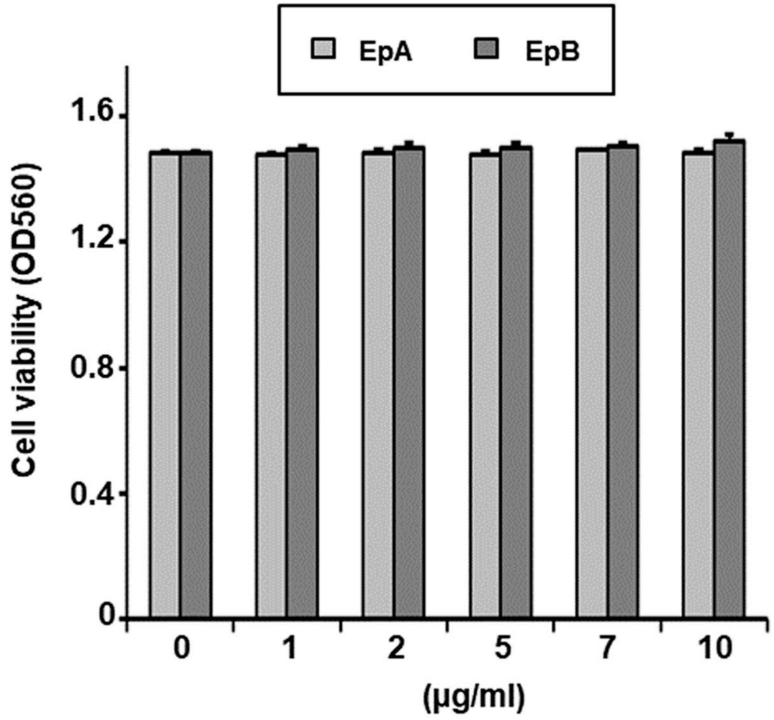

Figure S7. Cytotoxicity of ethyl pheophorbides a and b against MDCK cells

MDCK cells were treated with different concentrations of ethyl pheophorbide a or b (EpA and EpB, respectively). At 48 h after treatment, cell viability was determined with the MTT assay. Experiments were performed in triplicate.
